# The Apoptotic Volume Decrease Is an Upstream Event of MAP Kinase Activation during Staurosporine-Induced Apoptosis in HeLa Cells

**DOI:** 10.3390/ijms13079363

**Published:** 2012-07-24

**Authors:** Yuichi Hasegawa, Takahiro Shimizu, Nobuyuki Takahashi, Yasunobu Okada

**Affiliations:** Department of Cell Physiology, National Institute for Physiological Sciences, Okazaki 444-8585, Japan; E-Mails: y_hasegawa@nskw.co.jp (Y.H.); takshimi@pha.u-toyama.ac.jp (T.S.); nobu@kais.kyoto-u.ac.jp (N.T.)

**Keywords:** apoptosis, MAP kinase, anion channel, shrinkage, volume regulation

## Abstract

Persistent cell shrinkage, called apoptotic volume decrease (AVD), is a pivotal event of apoptosis. Activation of the volume-sensitive outwardly rectifying Cl^−^ channel (VSOR) is involved in the AVD induction. On the other hand, activation of the MAP kinase (MAPK) cascade is also known to play a critical role in apoptosis. In the present study, we investigated the relationship between the AVD induction and the stress-responsive MAPK cascade activation during the apoptosis process induced by staurosporine (STS) in HeLa cells. STS was found to induce AVD within 2–5 min and phosphorylation of c-Jun *N*-terminal kinase (JNK) and p38 MAPK after over 20–30 min. VSOR blockers suppressed not only STS-induced AVD but also phosphorylation of JNK and p38 as well as activation of caspase-3/7. Moreover, a p38 inhibitor, SB203580, and a JNK inhibitor, SP600125, failed to affect STS-induced AVD, whereas these compounds reduced STS-induced activation of caspase-3/7. Also, treatment with ASK1-specific siRNA suppressed STS-induced caspase-3/7 activation without affecting the AVD induction. Furthermore, sustained osmotic cell shrinkage *per se* was found to trigger phosphorylation of JNK and p38, caspase activation, and cell death. Thus, it is suggested that activation of p38 and JNK is a downstream event of AVD for the STS-induced apoptosis of HeLa cells.

## 1. Introduction

The apoptosis process includes whole-cell shrinkage, activation of cysteine proteases called caspases, chromatin condensation, genome DNA fragmentation, and apoptotic body formation. It has been shown that the apoptotic volume decrease (AVD), an early essential component of apoptotic cell death, is driven by osmolyte efflux resulting mainly from activation of K^+^ and Cl^−^ conductance [1,2]. Thus, cell volume changes have been used as one of the key discriminators between apoptosis and necrosis, which are associated with persistent whole-cell shrinkage and swelling, respectively.

Cell volume regulation is an essential function for animal cells, because volume changes are coupled to a variety of physiological processes, such as cell proliferation, differentiation, migration and cell death [3–5]. The regulatory volume decrease (RVD) observed soon after cell swelling is accomplished by parallel activation of multiple types of K^+^ channels [3,5] and a specific type of swelling-activated Cl^−^ channel called volume-sensitive outwardly rectifying anion channel (VSOR) in numerous cell types [6,7]. On the other hand, non-swelling-coupled activation of VSOR has been reported to cause AVD under apoptotic conditions [8,9]. In HeLa cells, a bacterial alkaloid staurosporine (STS), which is an activator of the mitochondrion-mediated apoptotic pathway, was found to induce VSOR activation through the generation of reactive oxygen species (ROS), thereby leading to AVD [10]. However, the detailed signaling pathway for VSOR activation and AVD induction has not been clarified as yet [11].

Mitogen-activated protein (MAP) kinases (MAPKs), which are the family of kinases transducing signals from the cell membrane to the nucleus in response to a wide range of stimuli including stress, are known to be involved in apoptotic cell death [12]. Especially, apoptosis signal-regulating kinase 1 (ASK1), a member of MAP kinase kinase kinase (MAPKKK) family, as well as stress-responsive MAPKs including c-Jun *N*-terminal kinase (JNK) and p38 MAPK are activated in response to a variety of apoptotic stimuli [13–15]. Phosphorylation of ASK1 is involved in apoptosis [13–17] by inducing activation of JNK and p38 MAPK [14,18]. Also, it has been reported that inhibition of p38 and/or JNK suppresses the apoptotic events in various types of cells [19–21] including HeLa cells [22]. However, how MAPKs are involved in induction of AVD has not been elucidated.

In the present study, we first attempted in identifying the MAPK signaling pathway of the STS-induced apoptosis in HeLa cells. We then focused to determine whether AVD is a downstream or upstream event of STS-induced MAPK activation. Our results show that VSOR blockers reduced not only AVD induction but also phosphorylation of stress-responsive MAPKs induced by STS, but the suppression of these MAPKs did not impede AVD. In addition, we found that persistent cell shrinkage *per se* induced phosphorylation of stress-responsive MAPKs. Thus, it is concluded that AVD or persistent cell shrinkage precedes activation of stress-responsive MAPK activation in HeLa cells undergoing STS-induced apoptosis.

## 2. Results

### 2.1. VSOR Blocker Sensitivity of STS-Induced AVD and Phosphorylation of p38 and JNK

**Figure 1:**
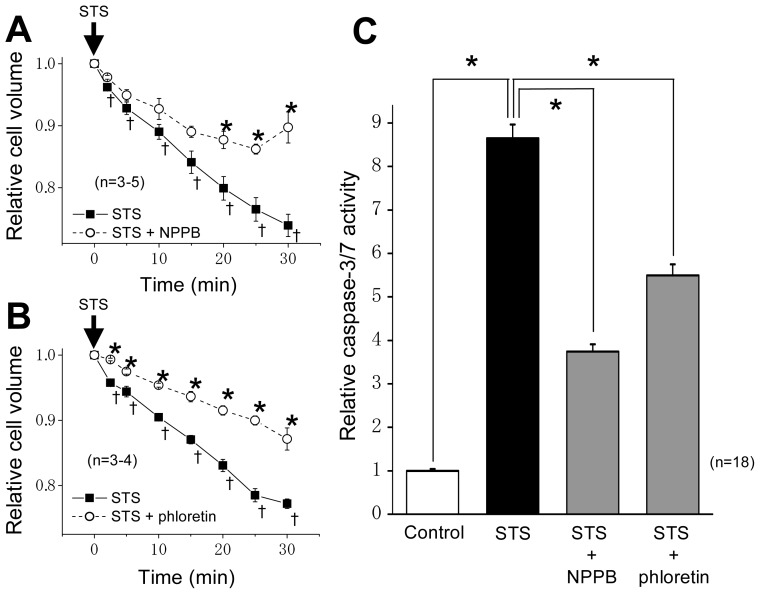
Sensitivity of staurosporine (STS)-induced apoptotic volume decrease (AVD) and caspase activation to volume-sensitive outwardly rectifying Cl^−^ channel (VSOR) blockers. The experiments with a VSOR blocker (200 μM 5-nitro-2-(3- phenylpropylamino)-benzoic acid (NPPB) or 100 μM phloretin) were performed in HeLa cells by adding either blocker to the bathing solution after pretreating the cells with either blocker for 1 h. (**A**, **B**) Time course of changes in the mean cell volume after stimulation with 4 μM STS in the absence (filled squares) or presence (open circles) of NPPB (**A**) or phloretin (**B**). Each symbol represents the relative mean cell volume at a given time (normalized by the mean cell volume at time zero), and each vertical bar represents the SEM value (*n* = 3–5). The mean cell volume (of around 3.3 pL) at time zero was not significantly different among experiments. * *p* < 0.05 *versus* the data in the absence of a VSOR blocker at a given time. ^†^ *p* < 0.05 between the data in the absence of a VSOR blocker at time zero and at a given time. (**C**) Caspase-3/7 activity before (Control: open column) or after 4-h stimulation with 4 μM STS in the absence (filled column) or presence of NPPB or phloretin (shadowed columns). Each column represents the relative mean value (normalized by the control value) with SEM (vertical bar) (*n* = 18). * *p* < 0.05 between two data designated.

Our previous study showed that VSOR blockers, 5-nitro-2-(3-phenylpropylamino)-benzoic acid (NPPB) and phloretin, suppress STS-induced AVD observed after ≥30 min and subsequent apoptotic biochemical events in HeLa cells [1,23]. As shown in Figure 1, not only STS-induced activation of caspase-3/7 observed after 4 h but also the AVD process observed as early as 2 to 5 min after STS stimulation were found to be significantly suppressed by NPPB (200 μM) and phloretin (100 μM) which are known to inhibit VSOR currents by 80~90% at these concentrations [24,25]. In the absence of STS, cell volume remained constant for over 30 min (data not shown), as observed previously [1].

**Figure 2:**
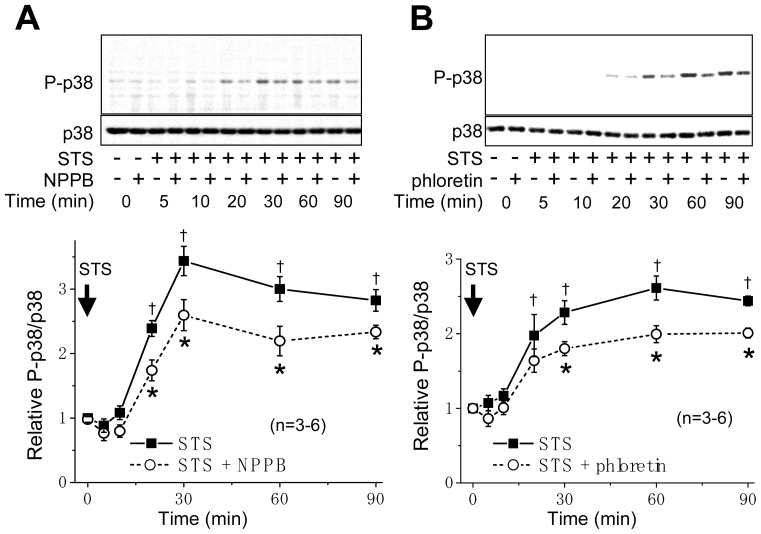
Sensitivity of STS-induced phosphorylation of p38 to a VSOR blocker, NPPB or phloretin, in HeLa cells. Top panels: Western blot analysis for time-dependent changes in the levels of phosphorylated p38 (P-p38) and non-phosphorylated p38 (p38) in the absence (−) or presence (+) of 200 μM NPPB (**A**) or 100 μM phloretin (**B**) without or with STS (4 μM) stimulation. Bottom panels: Time course of the ratio of P-p38 to p38 after STS stimulation in the absence (filled squares) or presence (open circles) of 200 μM NPPB (**A**) or 100 μM phloretin (**B**). Each symbol represents the relative mean value (normalized by the mean value at time zero) with SEM (vertical bar) (*n* = 3–6). * *p* < 0.05 *versus* the data in the absence of a VSOR blocker at a given time. ^†^ *p* < 0.05 between the data in the absence of a VSOR blocker at time zero and at a given time.

**Figure 3:**
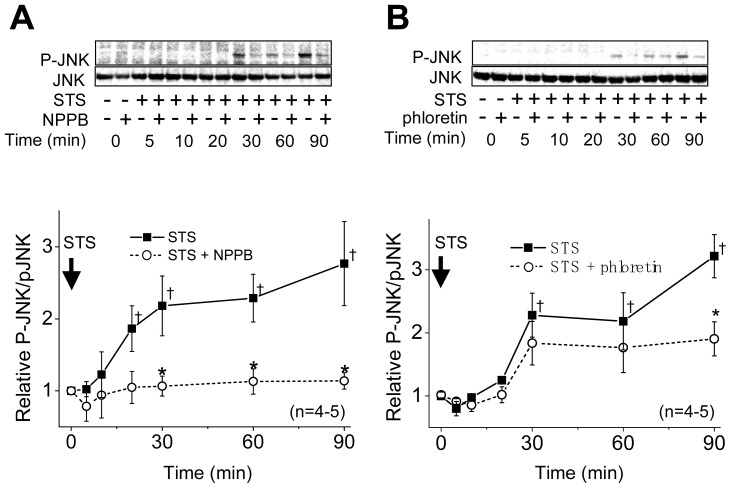
Sensitivity of STS-induced phosphorylation of JNK to a VSOR blocker, NPPB or phloretin, in HeLa cells. Top panels: Western blot analysis for time-dependent changes in the levels of phosphorylated JNK (P-JNK) and non-phosphorylated JNK (JNK) in the absence (−) or presence (+) of 200 μM NPPB (**A**) or 100 μM phloretin (**B**) without or with STS (4 μM) stimulation. Bottom panels: Time course of the ratio of P-JNK to JNK after STS stimulation in the absence (filled squares) or presence (open circles) of 200 μM NPPB (**A**) or 100 μM phloretin (**B**). Each symbol represents the relative mean value (normalized by the mean value at time zero) with SEM (vertical bar) (*n* = 4–5). * *p* < 0.05 *versus* the data in the absence of a VSOR blocker at a given time. ^†^ *p* < 0.05 between the data in the absence of a VSOR blocker at time zero and at a given time.

The STS treatment significantly increased phosphorylation of p38 at ≥20 min (Figure 2). However, in the presence of a VSOR blocker, NPPB or phloretin, phosphorylation of p38 became less marked in comparison with that in the absence of the VSOR blocker (Figure 2). An increase in the JNK phosphorylation was also observed at ≥20–30 min after stimulation with STS (Figure 3). The STS-induced phosphorylation of JNK was suppressed by these VSOR blockers (Figure 3). In the absence of STS, both NPPB and phloretin affected neither the levels of phosphorylated p38 and JNK nor those of non-phosphorylated ones up to 180 min after application (data not shown).

**Figure 4:**
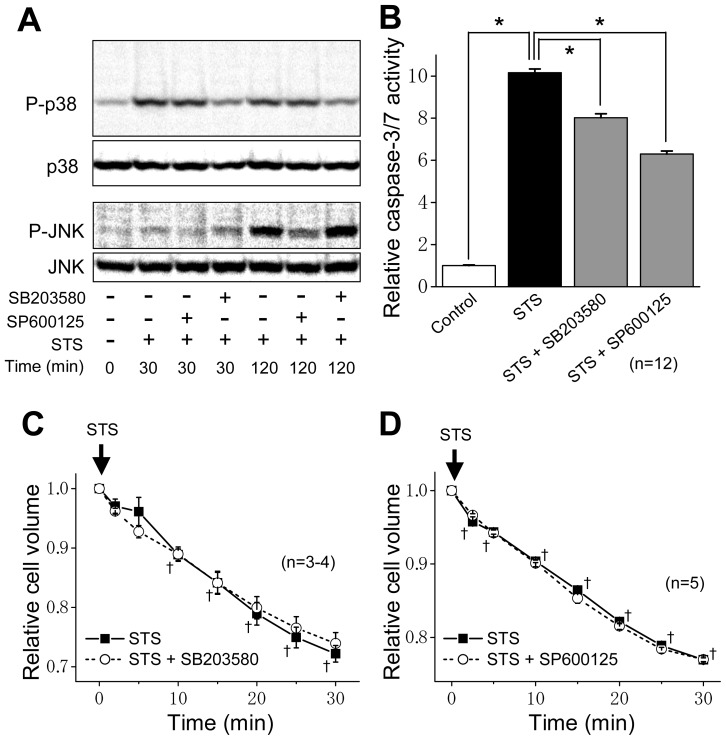
Effects of a MAPK inhibitor on STS-induced MAPK phosphorylation, caspase activity and AVD in HeLa cells. (**A**) Western blot analysis for the levels of P-p38, p38, P-JNK and JNK in the absence (−) or presence (+) of 10 μM SB203580 or 15 μM SP600125 30 min or 120 min after STS (4 μM) stimulation. (**B**) Caspase-3/7 activity before (Control: open column) or after 4-h stimulation with 4 μM STS in the absence (filled column) or presence of 10 μM SB203580 or 15 μM SP600125 (shadowed columns). Each column represents the relative mean value (normalized by the control value) with SEM (vertical bar) (*n* = 12). * *p* < 0.05 between two data designated. (**C**, **D**) Time course of changes in the mean cell volume after stimulation with 4 μM STS in the absence (filled squares) or presence (open circles) of 10 μM SB203580 (**C**) or 15 μM SP600125 (**D**). Each symbol represents the relative mean cell volume at a given time (normalized by the mean cell volume at time zero), and each vertical bar represents the SEM value (*n* = 3–5). ^†^ *p* < 0.05 between the data in the absence of a MAPK inhibitor at time zero and at a given time.

Since the AVD induction preceded activation of p38 and JNK, it is inferred that AVD is an upstream event of the MAPK activation. To testify this inference, the effects of MAPK inhibitors were examined. As shown in Figure 4A, a p38 MAPK inhibitor SB203580 [26] and a JNK MAPK inhibitor SP600125 [27] suppressed phosphorylation of p38 and JNK, respectively. These results are in accord with recent reports that not only catalytic activities of p38 and JNK but also phosphorylation of these MAPKs by upstream MAPK kinases are inhibited by SB203580 [28–30] and SP600125 [31–33], respectively, under certain conditions. These MAPK inhibitors also suppressed activation of caspase-3/7 induced by the STS treatment (Figure 4B). However, these MAPK inhibitors failed to suppress AVD induced by the STS treatment (Figure 4C,D). These results are in agreement with previous observations in salmonid hepatoma and gill cells stimulated with STS [34].

Taken together, it is suggested that the VSOR blockers reduce phosphorylation of stress-responsive MAPKs induced by apoptotic stimulation with STS and that the MAPK activation is a downstream event of VSOR-dependent AVD induction.

### 2.2. ASK1 Dependence of STS-Induced Caspase Activation but not of AVD

**Figure 5:**
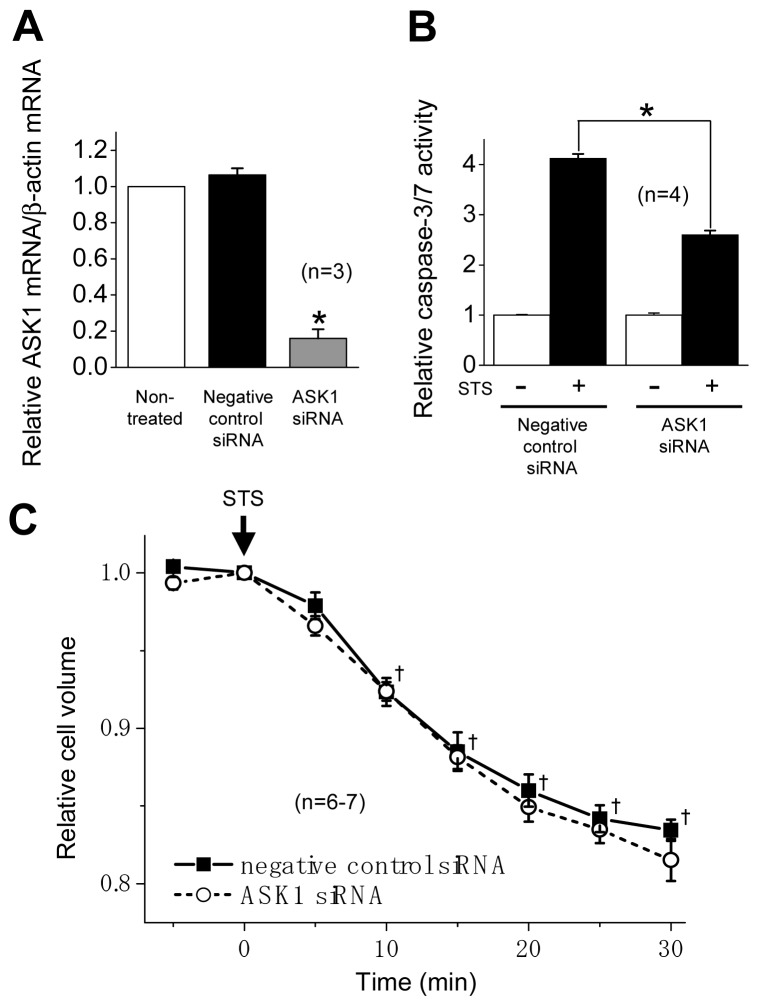
Effects of siRNA-mediated knockdown of ASK1 on the ASK1 mRNA level, STS-induced caspase activation and AVD in HeLa cells. (**A**) Down-regulation of ASK1 mRNA expression by 2-day treatment with ASK1 siRNA but not by that with negative control siRNA. Each column represents the relative mean ratio of ASK1 mRNA to β-actin mRNA (normalized by the non-treated control value) with SEM (vertical bar) (*n* = 3). (**B**) Caspase-3/7 activity after 6-h stimulation with 4 μM STS in the cells transfected with ASK1 siRNA and negative control siRNA. Each column represents the relative mean value (normalized by the mean value in the absence of STS) with SEM (vertical bar) (*n* = 4). * *p* < 0.05 between two data designated. (**C**) Time course of changes in the mean cell volume after stimulation with 4 μM STS in the cells transfected with ASK1 siRNA (open circles) or negative control siRNA (filled squares). Each symbol represents the relative mean value (normalized by the mean cell volume at time zero) with SEM (vertical bar) (*n* = 6–7). The data treated with ASK1 siRNA were not significantly different from those treated with negative control siRNA at any time points tested. ^†^ *p* < 0.05 between the negative control data at time zero and at a given time.

ASK1 is an upstream component of the stress-responsive MAP kinases that serve as pivotal regulators of the stress-induced apoptotic cell death [14,18]. Thus, there is a possibility that ASK1 activation is involved in STS-induced AVD as an upstream event. To examine this possibility, RNAi experiments were performed to knockdown ASK1 expression in HeLa cells. The cells transfected with ASK1-specific siRNA, but not negative control siRNA, showed a decrease in the mRNA level to 16% (Figure 5A). The transfection of ASK1-specific siRNA also partially suppressed the STS-induced caspase-3/7 activation (Figure 5B).

In contrast, as shown in Figure 5C, the STS-induced AVD in the cells transfected with ASK1-specific siRNA was not different from that in the negative control cells. These data suggest that ASK1 activity is involved in the STS-induced caspase-3/7 activation but not for the STS-induced AVD process in HeLa cells.

### 2.3. Induction of Phosphorylation of p38 and JNK by Sustained Cell Shrinkage

**Figure 6:**
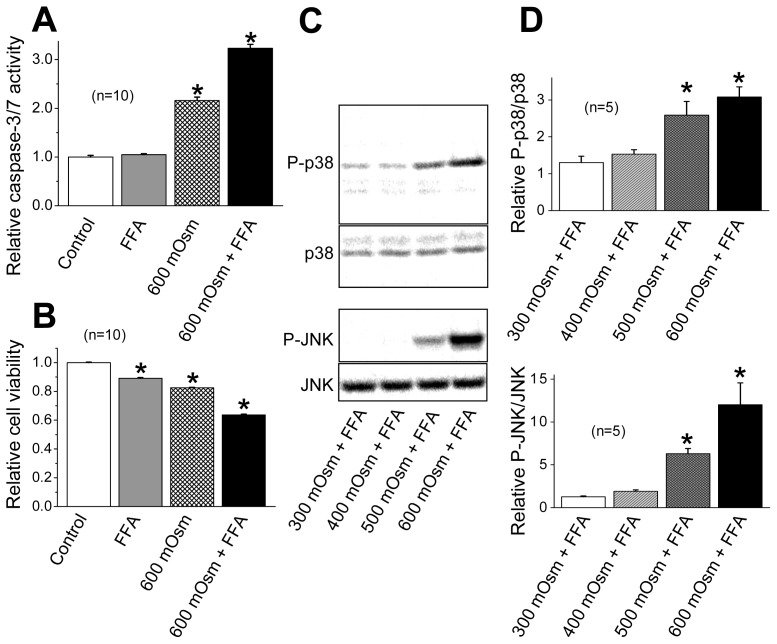
Effects of sustained osmotic shrinkage on caspase activity, cell death and MAPK phosphorylation in HeLa cells. (**A**, **B**) Caspase-3/7 activity and cell viability before (Control: open column) or after treatment with 100 μM FFA alone (shadowed column), 600 mOsmol/kg-H_2_O (mOsm) hypertonic solution (hatched column) or the hypertonic solution supplemented with 100 μM FFA (filled column) for 4 h (**A**) or 2 days (**B**). Each column represents the relative mean value (normalized by the control value) with SEM (vertical bar) (*n* = 10). * *p* < 0.05 *versus* Control. (**C**) Western blot analysis for osmolarity-dependent changes in the levels of P-p38 and P-JNK after incubation in isotonic (300 mOsm) or hypertonic (400, 500 or 600 mOsm) solution containing 100 μM FFA. (**D**) Ratios of P-p38 to p38 (top panel) and P-JNK to JNK (bottom panel) after incubation in 300, 400, 500 or 600 mOsm solution containing 100 μM FFA for 30 min. Each symbol represents the relative mean value (normalized by the control value at 300 mOsm in the absence of FFA) with SEM (vertical bar) (*n* = 3). * *p* < 0.05 *versus* the data for 300 mOsm + FFA.

**Figure 7:**
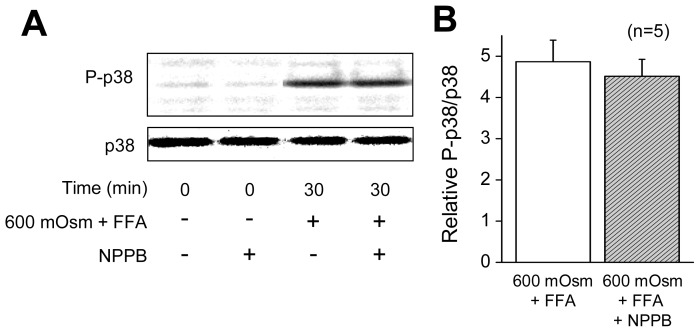
NPPB insensitivity of p38 phosphorylation induced by sustained osmotic cell shrinkage in HeLa cells. (**A**) Western blot analysis for changes in the levels of P-38 and p38 after incubation in hypertonic (600 mOsmol/kg-H_2_O) solution containing 100 μM FFA in the absence (−) or presence (+) of 200 μM NPPB for 30 min. (**B**) Ratio of P-p38 to p38 after incubation in 600 mOsmol/kg-H_2_O (mOsm) solution containing 100 μM FFA in the absence or presence of 200 μM NPPB. Each symbol represents the relative mean value (normalized by the control value at 300 mOsm in the absence of FFA) with SEM (vertical bar) (*n* = 5). * *p* < 0.05 *versus* the data without NPPB.

Since the above data suggest that AVD *per se* is a causal factor for activation of the stress-responsive MAPK cascade in HeLa cells, the effect of sustained cell shrinkage was next examined. The sustained cell shrinkage was induced by applying hypertonic stimulation (600 mOsmol/kg-H_2_O) in the presence of flufenamic acid (FFA), which blocks volume-regulatory hypertonicity-induced cation channel (HICC) thereby preventing the regulatory volume increase (RVI) [35,36]. Under hypertonic stress, addition of FFA resulted in more marked activation of caspase-3/7 after 4 h (Figure 6A) and a more prominent decrease in cell viability after 2 days (Figure 6B). Application of hypertonic stress together with FFA increased phosphorylation of p38 and JNK (Figure 6C,D), although FFA alone did not induce phosphorylation of p38 and JNK in the absence of hypertonic stimulation up to 180 min after application (data not shown). Phosphorylation of p38 and JNK was positively correlated with increases in osmolarity (Figure 6C,D). These results are in agreement with previous observations of these stress-responsive MAPK activation in association with osmotic shrinkage in a variety of cell types [37]. Moreover, hypertonicity-induced p38 phosphorylation was not significantly affected by a VSOR blocker (200 μM NPPB), as shown in Figure 7, consistently with a known fact that hypertonicity-induced cell shrinkage is a physical event without involving any volume-sensitive ion channels such as VSOR. These data indicate that the sustained cell shrinkage *per se* induces activation of the stress-responsive MAPKs.

## 3. Discussion

Apoptotic cell death is induced by the consequence of programmed chain reactions under physiological and pathophysiological conditions. Normotonic cell shrinkage, called AVD, is a major hallmark of apoptosis [1,2] and starts before caspase activation (at ≥30 min after apoptotic stimulation) in a variety of cell types [1,23]. In the present study, STS was found to induce AVD as early as 2 min after stimulation (Figure 1). The AVD induction is known to be triggered by activation of K^+^ and Cl^−^ conductances following stimulation with a mitochondrion-mediated or death receptor-mediated apoptosis inducer [1,2]. The VSOR activity, which is usually activated by cell swelling under non-apoptotic conditions [6,7], is known to be the major contributor to the AVD-inducing Cl^−^ conductance in a large variety of cell types [1,2,9–11]. In the present study, a VSOR blocker (phloretin or NPPB) actually suppressed the AVD event within 2–20 min after STS stimulation and then caspase-3/7 activation after 4 h (Figure 1).

Stress-responsive MAPKs (JNK and p38) are known to mediate apoptotic cell death [12]. In fact, simultaneous activation of p38 and JNK was observed in the apoptosis process under stimulation with a number of apoptotic stimuli [16,20,38]. In the present study, in HeLa cells, STS was found to induce phosphorylation of p38 (Figure 2) and JNK (Figure 3) as well as caspase-3/7 activation in a manner dependent of p38 and JNK activities (Figure 4). Also, a MAPKKK member, ASK1, which is involved in activation of p38 and JNK via phosphorylation of MAPK kinases (MKKs), was reported to be involved in apoptosis induced by various apoptotic stimuli [13–15,39]. The present study showed that it is also the case of STS-induced apoptosis in HeLa cells (Figure 5).

**Figure 8:**
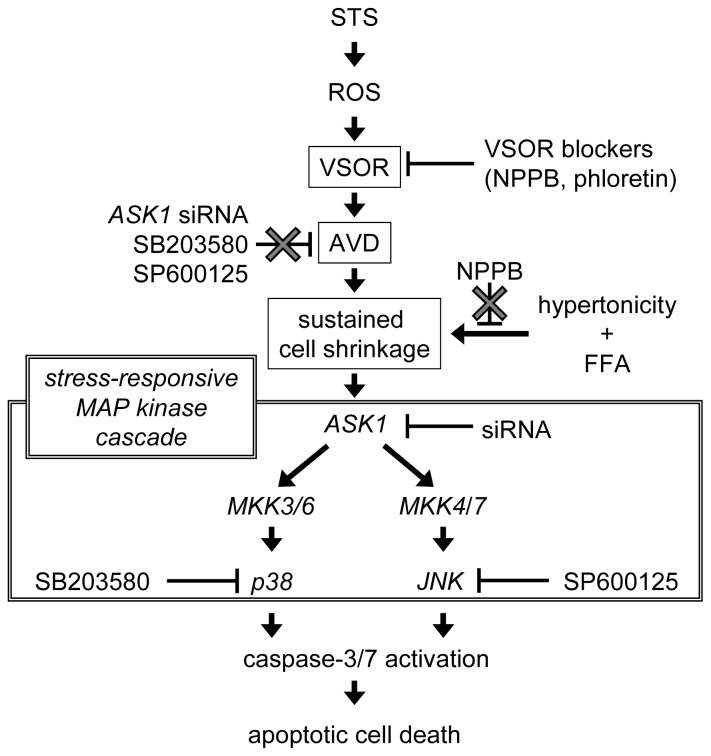
Proposed scheme of the cascade of apoptotic events including AVD and the stress-responsive MAP kinase cascade during the process of STS-induced apoptosis in HeLa cells.

Activation of p38 MAPK was reported to mediate activation of K^+^ channels involved in apoptosis in several cell types [40–42]. In contrast, it has been suggested that 4-aminopyridine-sensitive K^+^ channel activity is required for activation of p38 and JNK in myelocytic leukemic cells exposed to ultraviolet (UV) radiation [43]. Also, Cl^−^ efflux sensitive to a stilbene-derivative Cl^−^ channel blocker (SITS) was reported to play a role in activation of the MKK4-JNK cascade in UV-irradiated apoptotic Jurkat-T cells [44]. Therefore, there arises a question: Which is an upstream event, the AVD induction dependent on K^+^ and Cl^−^ conductances or activation of stress-responsive MAPKs, p38 and JNK? First, in the present study using HeLa cells, the STS-induced AVD induction was found to start much earlier than phosphorylation of p38 and JNK in a manner sensitive to VSOR blockers (Figure 1 *versus* Figures 2 and 3). Second, both a p38 inhibitor SB203580 and a JNK inhibitor SP600125 failed to affect the AVD process induced by STS (Figure 4). Third, siRNA-mediated knockdown of ASK1 also failed to suppress STS-induced AVD (Figure 5). Fourth, sustained osmotic cell shrinkage *per se* was found to induce phosphorylation of p38 and JNK in a manner dependent of hypertonicity (Figure 6) but in a manner insensitive to a VSOR blocker (Figure 7). On balance, it is inferred that AVD or sustained cell shrinkage is an upstream events of activation of stress-responsive MAPK cascade, as schematically depicted in Figure 8.

As suggested by Heimlich and Cidlowski [44], it is possible that Cl^−^ efflux may be a requisite for activation of stress-responsive MAPKs, because net Cl^−^ efflux was found to be coupled not only to STS-induced AVD but also to hypertonicity-induced cell shrinkage in HeLa cells [45]. Reactive oxygen species (ROS) are known to mediate STS-induced VSOR activation in HeLa cells [10]. ROS were also shown to mediate activation of VSOR without coupling to swelling in mouse astrocytes upon stimulation with exogenous application of bradykinin [46,47] or ATP [48]. This receptor-mediated ROS generation induced by bradykinin or ATP was demonstrated to involve protein kinase C, Ca^2+^ nanodomains and NADPH oxidases (NOX) [47,48]. However, the mechanisms by which ROS activate the VSOR activity leading to AVD remain to be elucidated.

## 4. Experimental Section

### 4.1. Chemicals and Antibodies

NPPB, phloretin, STS, and FFA were obtained from Sigma (St. Louis, MO, USA), and SB203580 and SP600125 were obtained from Calbiochem (San Diego, CA, USA). These chemicals were dissolved in DMSO to prepare stock solutions and applied by 1000 times dilution of these stock solutions.

Anti-phospho-p38 (Thr180/Tyr182), anti-phospho-JNK (Thr183/Tyr185), anti-p38 and anti-JNK primary antibodies, and anti-rabbit IgG HRP-linked secondary antibody were obtained from Cell Signaling Technology (Danvers, MA, USA).

### 4.2. Cell Culture and Apoptosis Induction

Human cervix HeLa cells were grown as a monolayer in Eagle’s minimum essential medium (MEM) supplemented with 10% fetal bovine serum, 40 IU/mL penicillin G, and 100 μg/mL streptomycin under humidified conditions with 95% air/5% CO_2_ at 37 °C. To induce apoptosis, HeLa cells in the log-growing phase were treated with 4 μM STS, as described previously [1] or hyperosmotic MEM (400 to 600 mOsmol/kg-H_2_O) supplemented with 100 μM FFA, as reported previously [30]. FFA was added to sustain hypertonicity-induced cell shrinkage, because FFA blocks HICC, which plays an essential role in the cell volume recovery process after osmotic cell shrinkage called RVI [29]. The osmolarity was adjusted by adding mannitol using a freezing-point depression osmometer (OM802; Vogel, Giessen, Germany).

### 4.3. Cell Viability Measurements

Cell viability of HeLa cells was assessed by measuring mitochondrial succinate dehydrogenase activity using Cell Counting Kit-8 (Dojindo, Kumamoto, Japan) according to the manufacturer’s instructions. Briefly, cells are placed in 96-well plates and cultured under humidified conditions with 95% air/5% CO_2_ at 37 °C with isotonic or hypertonic MEM (300 or 600 mOsmol/kg-H_2_O) in the absence or presence of 100 μM FFA for 2 days. Next, the Cell Counting Kit-8 solution was added to each well, and the sample was then incubated in humidified conditions with 95% air/5% CO_2_ at 37 °C for 2 h. Then, absorbance of each well was measured at 450 nm.

### 4.4. Caspase-3/7 Activity Measurements

Caspase-3/7 activity in HeLa cells was measured using the Apo-ONE Homogeneous Caspase-3/7 Assay (Promega, Madison, WI) according to the manufacturer’s instructions. Briefly, HeLa cells were treated for 4 or 6 h with 4 μM STS or hypertonic MEM (600 mOsmol/kg-H_2_O) in the absence or presence of 100 μM FFA in 96-well plates in humidified conditions with 95% air/5% CO_2_ at 37 °C. After discarding the treatment solution, Apo-ONE Caspase-3/7 reagent was added to the wells and then gently mixed. The cells in the wells were incubated at room temperature (23–26 °C) for 3 h. Finally, fluorescence (Excitation, 485 nm; Emission, 538 nm) of each well was measured.

### 4.5. Cell Volume Measurements

Cell volume was measured at room temperature (23–26 °C) by an electronic cell sizing technique with a Coulter-type cell size analyzer (CDA-500; Sysmex, Kobe, Japan), as described previously [49]. The mean volume of the cell population was calculated from the cell volume distribution measured. Suspensions of spherical cells were prepared by mechanical detachment from the plastic substrate. The suspensions were cultured with agitation for 15–300 min in a CO_2_ incubator. The standard isotonic solution (310 mOsmol/kg-H_2_O) was made of 95 mM NaCl, 4.5 mM KCl, 1 mM MgCl_2_, 1 mM CaCl_2_, 5 mM HEPES, and 105 mM mannitol (pH 7.4).

### 4.6. Western Blotting

Proteins from homogenized HeLa cells were separated by 4%–20% gradient SDS-PAGE gels, and then transferred to PVDF membranes. The membranes were blocked with ECL Advance Blocking Agent (GE Healthcare, Buckinghamshire, UK) and incubated with 1:2000 dilution of primary antibody in the CanGetSignal solution 1 (TOYOBO, Osaka, Japan) at 4 °C overnight followed be incubation with HRP linked secondary antibody diluents (1:3000) in the CanGetSignal solution 2 (TOYOBO) at room temperature for 1 h. After incubating the membranes with ECL Advance Western Blotting Detection Kit, the protein bands were detected with Typhoon 9400 scanner (GE Healthcare). Densitometry values of more than three independent experiments were estimated using Image-J software (NIH, Bethesda, MD).

### 4.7. RNA Interference

RNA interference experiments were performed using human ASK1-specific siRNAs and a negative control siRNA (BlockIT: FITC-labeled short RNA with random sequence; Invitrogen, Carlsbad, CA), as previously described [17]. Briefly, transfection of each siRNA was performed using HiPerFect Transfection Reagent (Quiagen, Hilden, Germany) according to the manufacture’s protocol. Transfected cells were rested for 2 days before subjecting to further analysis. Over 80% of cells were found to exhibit FITC fluorescent. Knockdown efficiencies of RNA interference were examined by quantitative RT-PCR.

### 4.8. Quantitative RT-PCR

Total RNA was extracted from HeLa cells using Sepasol-RNA (Nacalai Tesque, Kyoto, Japan). First-strand cDNA was synthesized from the total RNA with Transcriptor Reverse Transcriptase (Roche, Mannheim, Germany). The resultant first-strand cDNA was used for quantitative RT-PCR procedure. Quantitative RT-PCR was performed using 7300 Real-Time PCR System (Applied Biosystems, Carlsbad, CA). The threshold cycle (CT) was determined as the fractional PCR cycle number at which the fluorescence emission passed the threshold level within the exponential region of the amplification curve by the Fast System Software (Applied Biosystems).

### 4.9. Statistical Analysis

The data, presented as means ± SEM, were statistically analyzed using the ANOVA with Bonferroni’s post hoc test and Student’s *t* test. Differences were considered significant when *p* was < 0.05.

## 5. Conclusions

In the present study, we investigated the link between two prerequisite events for apoptosis induction, sustained cell shrinkage called AVD and phosphorylation of stress-responsive MAP kinases, p38 and JNK, due to activation of ASK1. It is suggested that sustained cell shrinkage or AVD is an independent and upstream event of the stress-responsible MAP kinase cascade in the STS-induced apoptosis process in HeLa cells.
